# Dr. William Garner Sutherland: The Man Who Changed Osteopathy Forever

**DOI:** 10.7759/cureus.78071

**Published:** 2025-01-27

**Authors:** H V Sharath, Pratik Phansopkar, Moh'd Irshad Qureshi, Raghumahanti Raghuveer, Gurjeet Kaur

**Affiliations:** 1 Paediatric Physiotherapy, Ravi Nair Physiotherapy College, Datta Meghe Institute of Higher Education and Research (Deemed to be University), Wardha, IND; 2 Musculoskeletal Physiotherapy, Ravi Nair Physiotherapy College, Datta Meghe Institute of Higher Education and Research (Deemed to be University), Wardha, IND; 3 Neurophysiotherapy, Ravi Nair Physiotherapy College, Datta Meghe Institute of Higher Education and Research (Deemed to be University), Wardha, IND; 4 Physiotherapy, Ravi Nair Physiotherapy College, Datta Meghe Institute of Higher Education and Research (Deemed to be University), Wardha, IND

**Keywords:** biodynamic model osteopathy cranial field, breath of life, cranio-sacral osteopathy, historical vignettes, primary respiratory mechanism

## Abstract

William Garner Sutherland (1873-1954), an American osteopathic pioneer, created the theory of cranial osteopathy, which completely changed the osteopathic field. His groundbreaking theory postulated that the previously believed-to-be-immobile skull bones actually exhibited delicate, rhythmic motions that were essential to general health. At first, there was doubt about Sutherland's work, but with time and clinical success, cranial osteopathy was acknowledged as a separate and important area of osteopathic medicine. He popularized the idea of the "primary respiratory mechanism," emphasizing the dynamic interaction between membranes inside the skull, cerebrospinal fluid, and cranial bones. His methods, intended to address imbalances in this system, have been beneficial in the treatment of a wide range of ailments, including developmental abnormalities and headaches. Sutherland founded the Sutherland Cranial Teaching Foundation, and the organization continues to carry out its mission to preserve cranial osteopathy, thus perpetuating Sutherland's legacy. His contributions have left a lasting impression on osteopathic medicine and allied disciplines, guaranteeing that practitioners will continue to be influenced by his creative ideas and that patient outcomes will be enhanced.

## Introduction and background

The American osteopathic physician William Garner Sutherland (1873-1954) is recognized for inventing cranial osteopathy. Sutherland studied under the renowned osteopathic physician Dr. Andrew Taylor Still, and it was during this time that he became interested in the cranial bones. Sutherland made a significant contribution when he proposed that the skull's bones had some degree of movement rather than being fused, as was previously believed. He suggested that this movement was essential to general health, as the rhythmic motion of the cerebrospinal fluid. Cranial osteopathy is a subspecialty of osteopathic medicine that focuses on the head and its relationship to the body. This concept was groundbreaking when it was first proposed [1].

Assessing and treating the subtle mechanical and dynamic motions of the skull and their effects on the nervous system and general health are key components of cranial osteopathy. Thanks to Sutherland's study, practitioners have developed strategies to address imbalances in these areas, which they believe can help a variety of illnesses, from headaches to developmental problems in children. Osteopathic doctors all over the world continue to use cranial osteopathy as a result of his long-lasting contributions to the field of osteopathic medicine [1, 2].

## Review

Childhood and schooling

William Garner Sutherland was born on March 27, 1873, in Portage County, Wisconsin, in the United States. His early years were characterized by intense interest in the natural world and curiosity. Before enrolling in the American School of Osteopathy (ASO; now known as the A.T. Still University of Health Sciences) in Kirksville, Missouri, he completed his schooling in nearby schools. Dr. Andrew Taylor Still established the ASO, the first medical school devoted to the practice of osteopathic medicine, which stresses the body's healing [2].

Graduation life

Dr. Sutherland began his studies at the ASO in 1895. In 1898, he graduated at the age of 25. Being an exceptionally intelligent student, Dr. Sutherland made a discovery at the ASO that would alter not only his own life but also the lives of other patients, osteopaths, and other people [1].

Interest towards osteopathy

Under Dr. Still's guidance, Sutherland developed an intense fascination for the human skull's anatomy. He saw that the joints between the skull's bones, known as sutures, seemed to be made for mobility. Though the general consensus was that the adult cranial bones were immobile, this observation inspired him to formulate his later views. He dissected a skull and noticed how similar the articular surfaces of the bones were to a fish's gills, reminiscent of both motion and respiration, so he started studying osteopathy in the cranial realm. This prompted decades of more research, during which he made several discoveries regarding the human body in both health and disease. His theory that the "life and motion" cycle underpinning health is driven by an "involuntary mechanism" is still relevant today. He just stated that the principles of osteopathy also applied to the head, never claiming to have invented a new idea. Other osteopaths joined him in his later years to continue developing and studying this paradigm [2].

In the UK, cranial osteopathy, a term used to describe these theories regarding the body's involuntary mechanism, was initially introduced in the 1970s. A few British osteopaths studied the ideas and subtle procedures in the United States under the tutelage of osteopaths who had studied under Sutherland. They then instructed other osteopaths, and ultimately a college dedicated to the study of these concepts was established. It was named the Sutherland Cranial College of Osteopathy in honor of the man who founded osteopathy in the cranial sector. The college not only teaches Sutherland's work but also how his successors in osteopathy developed it. It is frequently astounding how precise Sutherland and Still were in their grasp of human health, even in light of recent scientific advances. The institution takes great satisfaction in providing current and relevant instruction, incorporating new findings and perspectives from a wide range of disciplines, including biophysics, molecular biology, and embryology [3].

Career path and osteopathic contributions

Sutherland started practicing osteopathy in 1900, following his graduation from the American School of Osteopathy. Like most osteopaths of the day, he concentrated on general health and the musculoskeletal system in his early practice. Sutherland's interest in the cranium bones, however, only grew. Sutherland started formulating his idea of cranial movement in the early 1900s, calling it the "primary respiratory mechanism." According to this view, the cerebrospinal fluid, the membranes inside the skull, and the cranial bones were all a part of a dynamic system of movement and function that was essential to good health. He thought that disruptions in this system could cause a number of health issues and that a patient's condition could be improved by resolving these disruptions [4].

Many of Sutherland's contemporaries were skeptical of his beliefs. It didn't seem possible that the cranial bones could shift, especially given how inflexible an adult skull looks. Sutherland persisted in his studies and clinical work, nevertheless, creating a number of methods intended for the use and treatment of cranial dysfunctions. Sutherland's "cranial rhythmic impulse," which he felt could be palpated by skilled practitioners, is one of his central theories. It is a delicate movement inside the cranial bones and cerebrospinal fluid. He discovered that by using a range of practical methods to address imbalances in this system, he could effectively cure ailments ranging from migraines to developmental abnormalities [4,5].

Even though Dr. Sutherland was successful in treating patients with a wide range of health issues, his work did not stop the outcry against it. The book "The Cranial Bowl," which Dr. Sutherland released in 1939 with the hopes of sharing his findings, was not only poorly received but also exposed him to criticism and derision from his own peers. Not to be deterred, Dr. Sutherland kept publishing his work, frequently using a pseudonym to shield himself from more criticism. Among his creations are "Bedside Technic," which he presented in 1929; "Skull Notions," a six-piece compilation; and "Cranial Membranous Articular Strains," an article that appeared in The Western Osteopath [6].

Dr. Sutherland's work was beginning to be recognized after more than 40 years, and more doctors were becoming aware of his "astounding" success in curing patients who had not responded to allopathic care (i.e., medications and/or surgery). Dr. Sutherland agreed to speak to the International Society of Sacroiliac Technicians in St. Louis in June 1940. Dr. Sutherland soon discovered that he was in high demand to speak to professional medical societies, train doctors to practice "manual medicine," answer consultation requests, and teach workshops. In 1944, Dr. Sutherland consented to instruct a postgraduate course at the Des Moines Still College of Osteopathy and Surgery on cranial osteopathy. In the middle of the 1940s, interest in Dr. Sutherland's study started to really take off. As a result, in 1946, the Osteopathic Cranial Association was founded. It is still the premier organization for his work and has a membership roster of doctors who are particularly committed to his work in the field of cranial osteopathy. The Cranial Academy is the current name of the organization [6, 7].

Personal life

Compared to his career accomplishments, William Garner Sutherland was a very private person and not much is known about his personal life. In 1906, he wed fellow osteopath Adah Strickler, and the two of them had one child. Adah supported William's work and made her own contributions to the field of osteopathy, demonstrating the Sutherlands' joint commitment to the medical profession. Sutherland was regarded for his kind disposition and commitment to his patients and students, even in the face of difficulties in getting his ideas accepted. Throughout his life, he practiced and taught osteopathic medicine despite criticism and hostility from the medical establishment. In spite of an increasing number of devoted pupils and grateful clients, Dr. Sutherland faced a barrage of criticism and was called a "quack" for his theories regarding the workings of "primary respiration." Due to the burden of those formative years, Dr. Sutherland's first marriage failed, and he divorced in the 1920s. In 1927, Dr. Sutherland got married again, and he lived out the rest of his days with Ada, his second wife. In 1939, cranial osteopathy was the subject of intense study. Today, scientific research and computer imaging technologies support Dr. Sutherland's observations, and his findings are acknowledged as true medical knowledge. For most of his life, Dr. Sutherland conducted research and worked as a physician in Missouri. At the age of 78, Dr. Sutherland relocated to Pacific Grove, California, in 1951, leaving the Midwest behind. He lived in California with his second wife, Ada Strand Sutherland, until his death in 1954. Osteopaths worldwide hold Dr. Sutherland in high regard for the priceless contribution he made to medicine [1,2].

Achievements and legacy

One of the most important developments in osteopathic medicine is the work of William Garner Sutherland in cranial osteopathy. After some time, his theories were accepted, and cranial osteopathy was acknowledged as a specialty in the area. Sutherland taught numerous students who went on to become well-known osteopathic practitioners in their own right, so his influence reached well beyond his own practice. To regulate the teaching of cranial procedures, Sutherland established the Sutherland Cranial Teaching Foundation in 1948. Sutherland's legacy will live on thanks to this institution, which keeps up the promotion and advancement of cranial osteopathy research [8]. The development of craniosacral therapy, a related area that employs similar ideas but is practiced by a larger spectrum of healthcare professionals, such as chiropractors and physical therapists, was made possible in part by Sutherland's work. Many ailments, such as headaches, migraines, temporomandibular joint disorders (TMJ), and developmental delays in children, have been treated with his approaches. A medal granted by the Minnesota State Osteopathic Association for exceptional achievement, honorary lifelong membership in this organization, Academy of Applied Osteopathy Yearbook Honoring Dr. William G. Sutherland, the Academy of Applied Osteopathy gave a lifelong honorary membership and an honor medal; the Kirksville College of Osteopathy and Surgery bestowed onto its honorary doctorate, Doctor of Osteopathic Sciences [1].

## Conclusions

In the discipline of osteopathic medicine, William Garner Sutherland's groundbreaking work in cranial osteopathy has left a lasting impression. Numerous patients have found comfort in his inventive treatment methods and his unwavering commitment to comprehending the intricate mechanics of the human skull, which has also served as an inspiration to future generations of osteopaths. Despite initial resistance, his concepts are now fundamental to osteopathic practice, and his imprint is still felt in the profession today.
